# Molecular Modifications of the Pseudomonas Quinolone Signal in the Intermicrobial Competition with *Aspergillus*

**DOI:** 10.3390/jof7050343

**Published:** 2021-04-28

**Authors:** Hasan Nazik, Gabriele Sass, Paul Williams, Eric Déziel, David A. Stevens

**Affiliations:** 1 Infectious Diseases Research Laboratory, California Institute for Medical Research, San Jose, CA 95128, USA; hasannazik01@gmail.com (H.N.); gabriele.sass@cimr.org (G.S.); 2 Biodiscovery Institute and School of Life Sciences, University of Nottingham, Nottingham NG7 2RD, UK; Paul.Williams@nottingham.ac.uk; 3 Centre Armand-Frappier Santé Biotechnologie, Institut National de la Recherche Scientifique (INRS), Laval, QC H7V 1B7, Canada; eric.deziel@iaf.inrs.ca; 4 Division of Infectious Diseases and Geographic Medicine, Stanford University Medical School, Stanford, CA 94305, USA

**Keywords:** *Aspergillus*, *Pseudomonas*, Pseudomonas quinolone signal, intermicrobial competition

## Abstract

The Pseudomonas quinolone signal (PQS) is an important quorum-sensing molecule for *Pseudomonas aeruginosa* that regulates virulence factors, chelates iron, and is an important factor in interactions with eukaryotes, including fungi and mammalian hosts. It was previously shown to inhibit or boost *Aspergillus*, depending on the milieu iron concentration. We studied several molecular modifications of the PQS molecule, and their effects on *Aspergillus* biofilm metabolism and growth in vitro, and the effects of iron supplementation. We found that most molecules inhibited *Aspergillus* at concentrations similar to that of PQS, but with relatively flat dose-responses, and all were less potent than PQS. The inhibition was reversible by iron, suggesting interference with fungal iron metabolism. Stimulation of *Aspergillus* was not noted. We conclude that the critical *Aspergillus*-inhibiting moeities of the PQS molecule were partially, but not completely, interfered with by molecular modifications at several sites on the PQS molecule. The mechanism, as with PQS, appears to relate to fungal iron metabolism.

## 1. Introduction

*Pseudomonas aeruginosa* (Pa) and *Aspergillus fumigatus* (Af) encounter each other in nature in soil and water, and are, in most studies, the most frequently found bacterium and fungus in the airways of immunocompromised hosts, including persons with cystic fibrosis (CF) [1,2]. Thus, it is expected that these two microbes likely compete for their establishment and growth in various milieu. This has been studied extensively in vitro, and to a lesser extent in animal models, a subject reviewed in detail elsewhere [3,4,5,6,7,8,9].

Among the molecules identified in the intermicrobial competition is 2-heptyl-3-hydroxy-4(1*H*)-quinolone, the Pseudomonas Quinolone Signal (PQS). PQS is a quorum-sensing molecule, coordinating many Pa functions [8,10]. It chelates iron (Fe) and delivers it to the bacterial cell membrane in conjunction with Pa siderophores [10,11,12,13]. It upregulates genes involved in Pa oxidative stress responses, but can also be a pro-oxidant and induce oxidative stress; it regulates Pa virulence factors, and in intermicrobial interactions it can be used as a quorum sensing signal by other bacterial species, inhibit the respiratory chain, or induce membrane vesicle formation in other bacteria; and it can be inactivated by other bacteria [14,15,16]. Relevant to eukaryotes, PQS production can be inhibited by fungi, and in contrast, PQS can inhibit fungal biofilm formation [14,15,17,18]. In eukaryotic hosts, PQS can be toxic to host cells, and impair host defenses [12,14,19]. Its probable relevance in vivo is heightened by reports of its presence in body fluids of patients [12,14].

In a previous study [18], we focused on PQS inhibition of Af biofilm metabolism in a low Fe environment (RPMI-1640 medium), and in ancillary experiments, PQS inhibited Af growth on agar as well. The biofilm inhibition was reversed by the addition of Fe. Most remarkably, and paradoxically, in an enriched Fe environment, PQS + Fe boosted Af biofilm metabolism, as well as planktonic Af growth and growth on agar. This boosting exceeded the boosting effect of Fe alone. Perhaps uniquely biologically, the maximum boosting effect required Af siderophore involvement.

**Figure 1:**
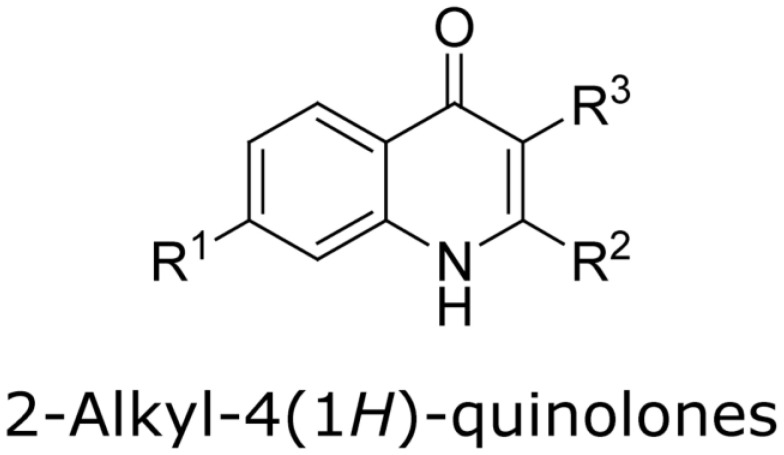
Molecules in this study. In all, R^1^ is an H atom. In Pseudomonas quinolone signal (PQS), 2-heptyl-4-hydroxyquinoline (HHQ), 2-heptyl-3-chloro-4(1*H*)-quinolone (3Cl-PQS) and 2-heptyl-3-amino-4(1*H*)-quinolone (3NH_2_-PQS) R^2^ is an alkyl side chain, C_7_H_15_. In C1-PQS, C3-PQS, C5-PQS, and C9-PQS R^2^ is an alkyl side chain of 1, 3, 5, and 9 carbon molecules, respectively. In PQS and HHQ, R^3^ is a hydroxyl group or an H atom, respectively. In 3Cl-PQS and 3NH_2_-PQS, R^3^ is a chlorine or primary amine group, respectively.

Research (with some interest in the possibility of generating inhibitors of Pa) in several laboratories, studying molecular modifications of the PQS molecule, has indicated a high degree of structural PQS specificity for its functions in Pa physiology [17,20,21]. We therefore studied whether such congeners would act similarly to PQS in the intermicrobial interactions described for PQS. The chemical formulae for the molecules studied are given in Figure 1 [20,22].

HHQ (2-heptyl-4-hydroxyquinoline) is a PQS precursor, and it also activates the transcriptional regulator PqsR (also known as MvfR) [20] (2-heptyl-3-amino-4(1*H*)-quinolone, 3NH_2_-PQS retains this capability). PqsR is the Pa protein that plays a central role in the alkyl quinolone-dependent quorum-sensing cascade, and is a regulator of multiple virulence factors (recently reviewed in [8]). The NH_2_ group is isosteric with OH, but would have opposite electronic effects on the bicyclic ring. 2-Heptyl-3-chloro-4(1*H*)-quinolone (3Cl-PQS) is unable to activate PqsR, and the 3-Cl substitution will have different molecular effects than the 3-OH on the bicyclic ring. Modifications of the 2-alkyl side chain, of all the substitutions, would be likely to retain Fe-chelating properties of PQS (in contrast to 3NH_2_-PQS and 3Cl-PQS that lack the adjacent oxygen molecules at the 3 and 4 positions on the molecule), but result in different hydrophobicities that could impact uptake into and/or export from cells and the interaction targets.

## 2. Methods

### 2.1. Reagents

RPMI (Roswell Park Memorial Institute) 1640 medium (also referred to subsequently as “RPMI”) was obtained from Lonza, Walkersville, MD, USA, who indicated that the formulation contains no added Fe. There are traces of Fe in that medium, but <1 μM (limit of detection) as determined by inductively coupled plasma optical emission spectroscopy (Paolo Visca, Rome, Italy; personal communication). PQS (dissolved in ethanol, diluted with RPMI medium to prepare a 10 mg/mL stock solution; further diluted for experiments, as detailed in Results), FeCl_3_, menadione (for the XTT assay, 2,3-bis-(2-methoxy-4-nitro-5-sulfophenyl)-2*H*-tetrazolium-5-carboxanilide) were obtained from Sigma-Aldrich, St. Louis, MO, USA. FeCl_3_ is referred to subsequently, in terms of the concentration of the Fe^+3^ ion, as “Fe”.

The other molecules studied were synthesized in the authors’ laboratories: HHQ by Prof. E. Déziel, and all the remaining molecules studied by Prof. P. Williams [20,22], who also synthesized 3Cl-PQS by similar methods [20,22]. All these molecules were dissolved in DMSO (dimethyl sulfoxide) from Sigma-Aldrich and diluted with RPMI medium to prepare 5 mg/mL stock solutions, further diluted for experiments, as detailed in Results (the exception was 3NH_2_-PQS, dissolved in ethanol, and diluted with RPMI medium to prepare a 10 mg/mL stock solution). The Pa-related molecules were prepared and diluted for study as μg/mL concentrations; the corresponding molecular weights and molarities are given in Table 1. A final concentration of 50 μg/mL was generally the highest concentration that could be cleanly tested, owing to solubility issues and reagent availability. For all agar studies, 100 mm diameter plastic Petri dishes were used (E and K Scientific, Santa Clara, CA, USA). Bacto agar used for growth studies was obtained from Carolina Biological Supply Co., Burlington, NC, USA.

### 2.2. Isolates

In determining activity with Af as target, we desired to have confirmation by demonstrations with two Af wild-types; AF293 (ATCC MYA-4609) and 10AF (ATCC 90240). The former is a widely distributed reference strain, that has been sequenced [23], the latter is a virulent patient isolate [24,25]. Af stocks for long term storage are frozen at −80 °C in PBS (phosphate-buffered saline) with 0.2% Tween with 10% glycerol, in Microbank microbial storage vials (Pro-Lab Diagnostics, Richmond Hill, ON, Canada).

### 2.3. Assays

Measurement of inhibition of Af forming biofilm, and preformed Af biofilm, on plastic surfaces using the XTT assay, was as previously described [26]; assays referring to biofilm formation were assayed after 16 h incubation, and assays referring to preformed biofilm were evaluated after 40 h incubation. XTT assays measure Af metabolism, although many publications have taken the results of such assays as a surrogate for microbial growth [27]. Final concentrations of ethanol or DMSO were ≤0.25%, which is well below the 1% that we have determined is the inert concentration of these solvents in experiments. In all experiments, 2–3 controls were studied: RPMI alone, plus ethanol and/or DMSO ≤0.25% in RPMI (depending on the diluents for the molecules studied). Results with the 3 controls did not differ, and for simplicity of figures, not all 3 controls are shown.

Studies under hypoxic conditions were performed using BD GasPak EZ Campy Pouch System (BD, Franklin Lanes, NJ, USA) to contain the plates as previously described, producing an atmosphere of 10% oxygen [28].

Growth assays on agar, one spot method: RPMI-Bacto agar, as previously described [26], was inoculated with 10 µL of Af suspension (0.5 × 10^4^/mL conidia in RPMI) in quadruplicate per plate. Ten µL of PQS, its congeners, or HHQ were added to the 10AF conidia spots at a final concentration of 12.5 μg/mL. Separate plates were used for each molecule to be tested. Final ethanol or DMSO concentrations alone in RPMI as stated above did not alter 10AF growth on agar. We therefore compared all test molecules in agar growth experiments to a pure RPMI control. Plates were kept at room temperature without movement until drops settled into the agar, assuring that fungal colonies would have the same area from which to spread (5 mm diameter). Plates were incubated at 37 °C for 48 h. To quantify effects on Af growth, diameters of colonies were determined, and the growth area was calculated (π times radius^2^).

### 2.4. Statistical Analysis

The software used was GraphPad Prizm 9.0.1 (GraphPad Software, San Diego, CA, USA). Results were analyzed with Student’s *t*-test if two groups were compared and one-way analysis of variance (ANOVA) combined with Tukey’s post-test for multiple comparisons. All data are expressed as the mean ± the standard deviation. Data reported as percentages of the control value (e.g., when data from replicate experiments were combined) were compared with Student’s *t*-test after arcsin transformation of the proportions; these data are presented as percentages. Biofilm assays used 4–8 replicate wells for each group studied, for statistical purposes. Representative experiments shown are the most complete of a series of experiments; the conclusions for corroborating experiments are given in the text. Each figure, in its parts, shows several different experiments, but each figure is focused on a particular molecule. Statistical significance was regarded as *p* < 0.05.

## 3. Results

### 3.1. Studies with 3Cl-PQS

**Figure 2:**
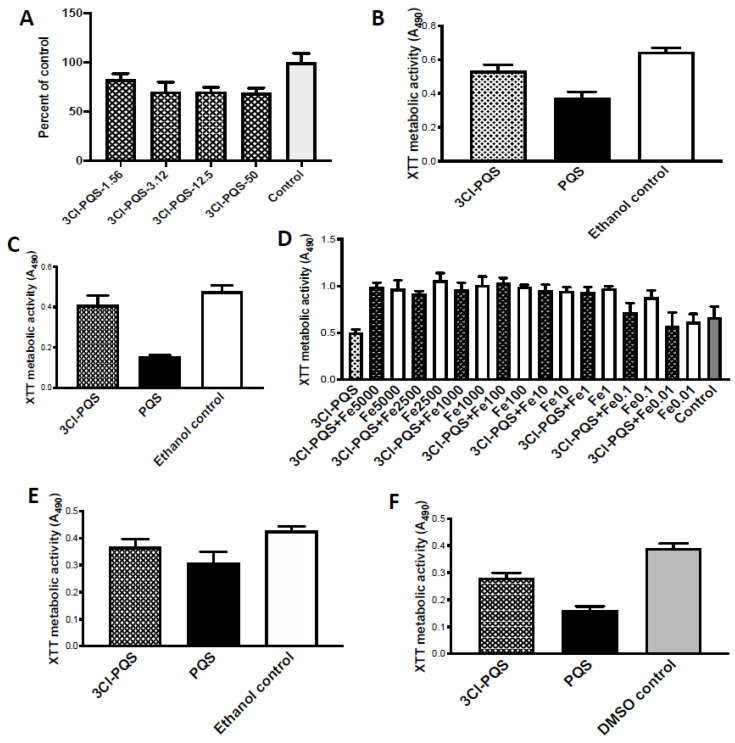
Studies with 3Cl-PQS on preformed Af biofilm metabolism, assessed by XTT assay. (**A**) Dose response study, strain 10AF, control (same amount of DMSO as in test articles). Designations 1.56, etc. are μg/mL. Dose-response is shallow; 1.56 μg/mL, *p* < 0.01 compared to control, and other concentrations *p* < 0.001 compared to control. (**B**,**C**), comparison to PQS, all at 1.56 μg/mL, and to ethanol control (shown) identical to DMSO control. 3Cl-PQS is less inhibitory than PQS. In (**B**), using 10AF, 3Cl-PQS is *p* < 0.01, and PQS *p* < 0.001, compared to control; PQS is *p* < 0.001 compared to 3Cl-PQS. In (**C**), studying strain Af293, 3Cl-PQS is *p* > 0.05, and PQS *p* < 0.001, compared to control; PQS is *p* < 0.001 compared to 3Cl-PQS. (**D**) Effect of Fe on 3Cl-PQS (12.5 μg/mL) inhibition of strain 10AF. Control (same amount of DMSO as in test article), numbers 0.01–5000 are Fe μM concentration. 3Cl-PQS inhibits Af metabolism (left bar, *p* < 0.01). Fe alone boosts Af metabolism (white bars), starting at Fe 0.1 μM, all *p* < 0.01–0.001 compared to control. Fe reverses 3Cl-PQS inhibition in combinations (black bars), beginning at Fe 0.01 μg/mL, where Fe 0.01 μM + 3Cl-PQS is now *p* > 0.05 compared to control or to 3Cl-PQS alone, and all other 3Cl-PQS + Fe combinations are greater (*p* < 0.001) compared to control or to 3Cl-PQS alone. 3Cl-PQS + Fe combinations do not boost Af metabolism above same concentration of Fe alone, unlike previously reported with PQS [18] (and at two concentrations, Fe 0.1 or Fe 2500 μM + 3Cl-PQS, are significantly, *p* < 0.05, less than corresponding Fe alone). (**E**,**F**), effects in hypoxic environment. DMSO and ethanol control results shown identical. (**E**) is Af isolate 10AF, (**F**) is Af isolate Af293, both tested at 1.56 μg/mL of test articles. In (**E**), 3Cl-PQS is *p* < 0.05 compared to control or to PQS, and PQS *p* < 0.001 compared to controls. In (**F**), PQS and 3Cl-PQS both *p* < 0.001 compared to controls, and PQS and 3Cl-PQS differ by *p* < 0.001.

Dose-response studies revealed a shallow dose-response. Two studies were performed, with preformed Af biofilm, with overlapping dose concentrations, and comparisons with controls are presented as % of respective controls (Figure 2A). The two studies encompassed two-fold dilutions from 50 to 0.19 μg/mL. The lowest concentration, 0.19 μg/mL, was not significantly different from control; 0.39–0.78 μg/mL showed inhibition, but *p* < 0.05 compared to control; 1.56 μg/mL (shown) indicated more inhibition (*p* < 0.01 compared to control); and 3.12 through 50 μg/mL were *p* < 0.001 compared to control, but none of these were significantly different (*p* > 0.05) from each other. The conclusion was a maximal effect at a low concentration, and higher concentrations could not increase that effect.

Two comparisons were made with PQS, all at 1.56 μg/mL compared to control (Figure 2B,C), with different Af strains. In both, PQS was a more potent inhibitor than 3Cl-PQS.

Studies were performed with a fixed concentration of 3Cl-PQS in the presence or absence of twofold dilutions of Fe (Figure 2D). Fe alone enhances Af metabolism. Fe (at concentrations as low as 0.01 μM) reverses inhibition by 3Cl-PQS. In contrast to what was found with PQS [18], 3Cl-PQS does not enhance Fe-mediated stimulation at high Fe concentrations, above that seen with Fe alone. These observations were confirmed in a second experiment, of identical design. (In these experiments, possibly partially incomplete solubilization was visually noted in some tubes with concentrations ≥100 μM Fe).

Two studies were made under hypoxic conditions, with the two Af strains, and comparison with PQS (Figure 2E,F). The findings were quite similar, with both reagents, to those in normoxic conditions (Figure 2B,C), and PQS was again the more potent inhibitor.

### 3.2. Studies with 3NH_2_-PQS

**Figure 3:**
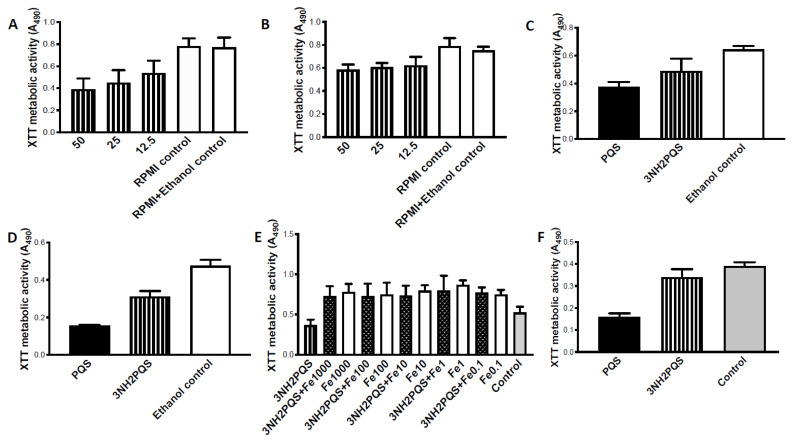
Studies with 3NH_2_-PQS. (**A**) Dose-response study with preformed 10AF biofilm. Minimal dose-response. “RPMI control” was biofilm without test article or diluent, “RPMI + Ethanol control” contained same concentration of ethanol diluent as present in test article wells; no difference between these controls. Both controls were also included in sections (**B**) through (**D**) of Figure 3, but as the results of these two controls was always the same, for simplification, only one control is shown in sections (**C**) through (**F**). Twofold dilutions of 3NH_2_-PQS were made from 50 to 0.78 μg/mL. Only at the very highest doses (12.5–50 μg/mL, shown) was there a suggestion of dose response, but there was no statistically significant difference among those bars. 3NH_2_-PQS at 0.78 μg/mL was not different from controls shown, and bars showing results with 1.56–6.25 μg/mL were not different from that of 12.5 μg/mL, so are not shown in order to simplify the figure. All dilution results from 1.56–50 μg/mL were *p* < 0.001 compared to controls. (**B**). Dose-response study with 10AF biofilm formation. Same experimental design as in A, but test against Af biofilm formation. Dilutions 0.78–6.25 μg/mL resulted in slight inhibition, but *p* > 0.05 compared to controls, and no dose-response. 3NH_2_-PQS 12.5 and 25 μg/mL *p* < 0.01 compared to controls, and 50 μg/mL *p* < 0.001 compared to controls. (**C**,**D**) Comparison with PQS, preformed biofilm, test reagents at 1.56 μg/mL. C, isolate 10AF. 3NH_2_-PQS is less potent inhibitor than PQS (*p* < 0.01). Both are *p* < 0.01 compared to controls. D, isolate Af293. PQS more inhibitory (*p* < 0.001) compared to 3NH2-PQS. Both are *p* < 0.001 compared to controls. (**E**) 3NH_2_-PQS (12.5 μg/mL) (left bar) inhibits preformed 10AF biofilm, *p* < 0.001 compared to control (right bar). Fe (0.1–1000 μM; numbers on x axis are these μM concentrations) reverses 3NH_2_-PQS inhibition (3NH_2_-PQS + Fe concentrations *p* < 0.001 compared to 3NH_2_-PQS). Fe boosts Af metabolism, but 3NH_2_-PQS + Fe does not increase metabolism above Fe alone. (**F**) Action of 3NH_2_-PQS under hypoxic conditions. 3NH_2_-PQS inhibitory to preformed Af293 biofilm (*p* < 0.05 compared to controls). PQS more inhibitory (*p* < 0.001) than controls, and more inhibitory compared to 3NH_2_-PQS (*p* < 0.001). Both PQS and 3NH_2_-PQS are at 1.56 μg/mL.

A two-fold dilution was made of 3NH_2_-PQS. The dose-response was minimal, with significant inhibiting activity starting at 1.56 μg/mL, and only the very highest doses testing showing incremental activity above that (Figure 3A).

Although all the present studies focused on preformed Af biofilm, one study, of similar design to the preceding, was performed with Af biofilm formation. The dose-response was similar in the latter study. Significant inhibition only occurred ≥12.5 μg/mL (Figure 3B).

Comparison with PQS was made with two Af strains (Figure 3C,D). With both, PQS was a significantly more potent inhibitor than 3NH_2_-PQS.

The ability to reverse inhibition with Fe was studied with Fe twofold dilutions (Figure 3E). Concentrations as low as ≥0.1 μM Fe reversed the 3NH_2_-PQS inhibitory effect on Af. The combination of Fe with 3NH_2_-PQS did not boost Af metabolism above that of Fe alone at the same concentration.

The inhibitory effect of 3NH_2_-PQS was also studied under hypoxic conditions, with Af isolate Af293 (Figure 3F). The effect was similar to that shown for normoxic conditions (Figure 3D), with PQS more potent. The results were the same (compare Figure 3C,F) in studies under hypoxia of isolate 10AF.

### 3.3. Studies of Alkyl Side Chains on PQS

**Figure 4:**
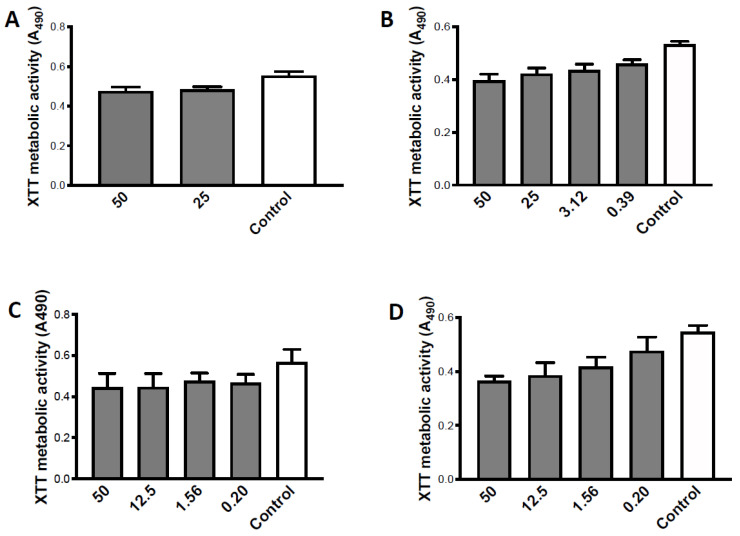
Studies of alkyl side chains on PQS. Dose-response assays. (**A**–**D**) are C1-PQS, C3-PQS, C5-PQS, C9-PQS respectively, tested against preformed 10AF biofilm, DMSO control shown. (**A**) Twofold dilutions C1-PQS 50 to 0.2 μg/mL were made. Dilutions 12.5 to 0.2 μg/mL produced slight and insignificant (*p* > 0.05) inhibition compared to control, and not different from each other (not shown). Only at 25 and 50 μg/mL was inhibition significant, and only at *p* < 0.05. (**B**) Same plan as in A, C3-PQS; 0.2 μg/mL produced slight and insignificant (*p* > 0.05) inhibition, and 0.39 to 50 μg/mL were all inhibitory compared to control (*p* < 0.01) and not significantly different from each other in effect. (**C**) Same plan as in A, C5-PQS. All dilutions were inhibitory (*p* < 0.05) compared to control, but were not different from each other. (**D**) Same plan as in A, C9-PQS. Slight dose-response, as shown. All dilutions were inhibitory compared to controls, but were not significantly different from each other; 0.2 to 0.78 μg/mL were *p* < 0.05 compared to controls, and 1.56 to 50 μg/mL were *p* < 0.001 compared to controls.

Dose-response studies were performed (Figure 4). Twofold dilutions of 50 to 0.2 μg/mL were made with the molecules with side chains of 1, 3, 5, or 9 length. With C1-PQS, significant inhibition was only seen with 25–50 μg/mL (Figure 4A). With C3-PQS, 0.39 μg/mL was significantly inhibitory, but higher concentrations were not more inhibitory (Figure 4B). C5-PQS was inhibitory at 0.2 μg/mL, but again higher concentrations were not more inhibitory (Figure 4C). C9-PQS was also inhibitory at 0.2 μg/mL, but there was an increment in activity ≥1.56 μg/mL. Overall, the dose-responses were shallow to non-existent.

**Figure 5:**
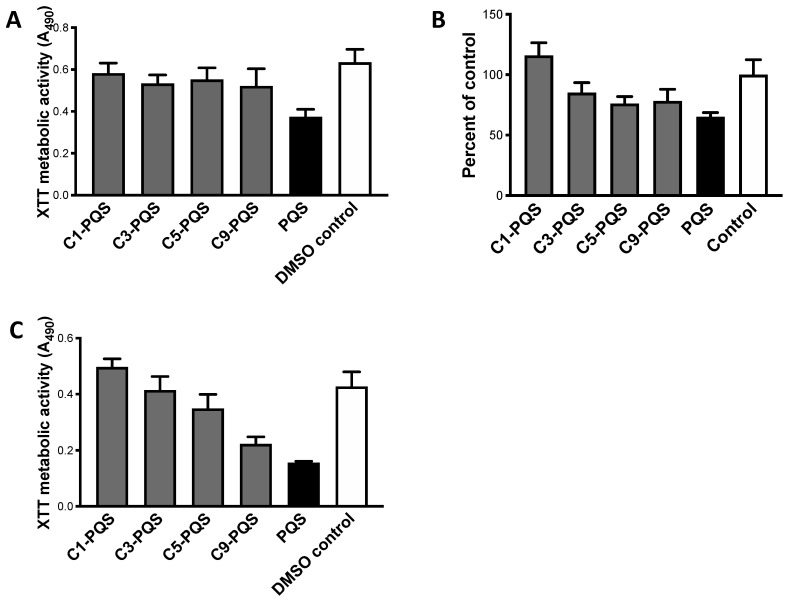
Studies of alkyl side chains on PQS. Comparisons of congeners with PQS. (**A**) Target 10AF, all molecules studied at 12.5 μg/mL. C1-PQS, C3-PQS, C5-PQS, C9-PQS, PQS respectively, are *p* > 0.05, *p* < 0.01, *p* < 0.05, *p* < 0.05, *p* < 0.001 compared to control. DMSO control results same as ethanol control shown. C1-PQS, C3-PQS, C5-PQS, C9-PQS are all *p* < 0.001 compared to PQS. (**B**) Target 10AF, all molecules studied at 1.56 μg/mL; separate plates, so data is referent to controls on each plate. DMSO controls shown, were same as ethanol and RPMI controls. C1-PQS, C3-PQS, C5-PQS, C9-PQS, PQS respectively, are *p* > 0.05 (actual boost above control), *p* < 0.01, *p* < 0.05, *p* < 0.05, *p* < 0.001 compared to control. C1-PQS, C3-PQS, C5-PQS, C9-PQS are *p* < 0.001, *p* < 0.001, *p* < 0.01, *p* < 0.01, respectively, compared to inhibition by PQS. (**C**) Target Af293, all molecules studied at 1.56 μg/mL. Shown are DMSO control same as ethanol control. C1-PQS, C3-PQS, C5-PQS, C9-PQS, PQS respectively, are *p* > 0.05 (actual boost above control), *p* > 0.05, *p* < 0.05, *p* < 0.001, *p* < 0.001 respectively, compared to control. C1-PQS, C3-PQS, C5-PQS, C9-PQS are all less inhibitory than PQS (*p* < 0.001).

These molecules were compared with PQS inhibitory activity (Figure 5).

When all molecules were studied at 12.5 μg/mL (Figure 5A) with isolate 10AF, C1 inhibition was not statistically significant, the other side chain variants were significantly inhibitory, and PQS was markedly more active compared to all of them. With all studied at 1.56 μg/mL (Figure 5B), C1-PQS was similar to the control, the others were inhibitory, and PQS was more strongly inhibitory than all of them. When the latter study was repeated with isolate Af293, C1-PQS and C3-PQS were noninhibitory. C5-PQS and C9-PQS were inhibitory, and this study produced the strongest suggestion that activity was related to chain length (Figure 5C), aside from PQS (chain length 7). PQS was again significantly more powerful.

**Figure 6:**
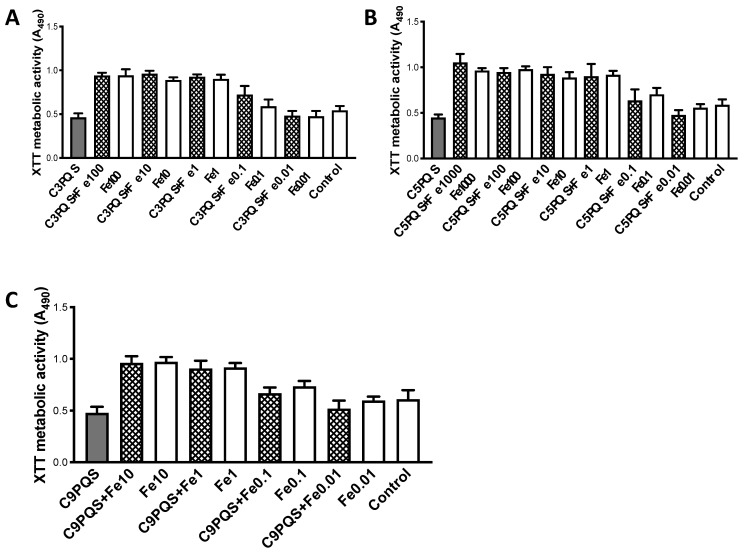
Studies of alkyl side chains on PQS. Fe reverses inhibition by side chain congeners, but combination of congeners with Fe does not boost metabolism above Fe alone. Target 10AF, all congeners studied at 12.5 μg/mL, DMSO controls are shown. Dilutions of Fe were made from 0.01 to 1000 μM final concentrations (these are the numbers on the x axis). In some experiments there were visible solubility problems with Fe 100–1000 μM, and the study values were the same as the next lower concentration; those bars are omitted. (**A**) C3-PQS (left bar) inhibits 10AF (*p* < 0.01) compared to control (right bar). Fe begins to reverse C3-PQS effect at 0.01 μM Fe, and with concentrations of Fe 100 μM with C3-PQS, are all at *p* < 0.001 compared to C3-PQS alone. No significant boosting across these concentrations above Fe alone, for C3-PQS + Fe. (**B**) C5-PQS (left bar) inhibits 10AF (*p* < 0.001) compared to controls (right bar). Fe evidently begins to reverse C5-PQS effect at 0.1 μM Fe, and at concentrations of Fe 1 to 1000 μM with C5-PQS, loss of inhibition is significant (*p* < 0.001) compared to C5-PQS alone. No significant boosting across these concentrations above Fe alone, for C5-PQS + Fe. (**C**) C9-PQS (left bar) inhibits 10AF (*p* < 0.01) compared to controls (right bar). Fe reverses C9-PQS effect at 0.1 to 10 μM Fe, loss of inhibition is significant (*p* < 0.001) compared to C9-PQS alone. No significant boosting across these concentrations above Fe alone, for C9-PQS + Fe.

With 10AF, reversal of inhibition by Fe was studied (Figure 6), with congeners at 12.5 μg/mL. C1-PQS was not studied, owing to the lack of inhibition in the absence of Fe. A concentration of Fe ≥0.01 μM significantly reversed C3-PQS inhibition (Figure 6A). An effect at that concentration was also seen with C5-PQS, and was significant at Fe ≥1 μM (Figure 6B). With C9-PQS, inhibition was also reversed with Fe ≥0.01 μM (Figure 6C).

Of note, with no congener + Fe was metabolism boosted above that seen with Fe alone at the same concentration (Figure 6A–C). This is different to the remarkable finding observed with PQS [18], where at high Fe concentrations, PQS combined with Fe could boost Af metabolism over that by Fe alone, in a manner dependent on the presence of Af siderophores.

**Figure 7:**
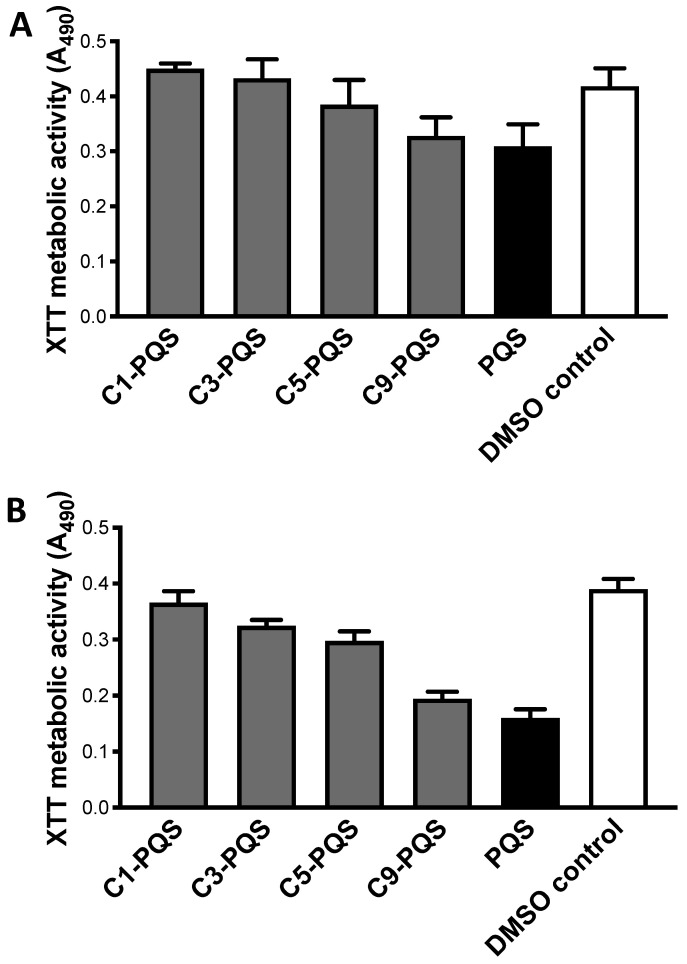
Studies of alkyl side chains on PQS. Effects in hypoxic environment. All molecules tested at 1.56 μg/mL. DMSO control results same as ethanol control. (**A**) Target 10AF. C1-PQS appears to boost slightly, compared to controls. C9-PQS and PQS inhibit significantly (both *p* < 0.001) compared to controls. PQS more inhibitory compared to C1-PQS, C3-PQS, C5-PQS, C9-PQS, respectively *p* < 0.001, *p* < 0.001, *p*< 0.05, *p* > 0.05. (**B**) Target Af293. C3-PQS, C5-PQS, C9-PQS, PQS all inhibit (*p* < 0.001) compared to controls, C1-PQS is *p* > 0.05 compared to controls. PQS more inhibitory compared to C1-PQS, C3-PQS, C5-PQS (*p* < 0.001 for all), and C9-PQS (*p* < 0.01).

Studies were performed in a hypoxic environment, with two Af strains, and congeners set at 1.56 μg/mL (Figure 7). The results (Figure 7) were not dissimilar to that seen in normoxic environments (Figure 5). PQS was again the strongest inhibitor of biofilm formation. Again, this pattern (Figure 7A,B) suggested that activity was directly related to chain length among congeners, aside from PQS itself (as in Figure 5C).

### 3.4. Studies with HHQ

**Figure 8:**
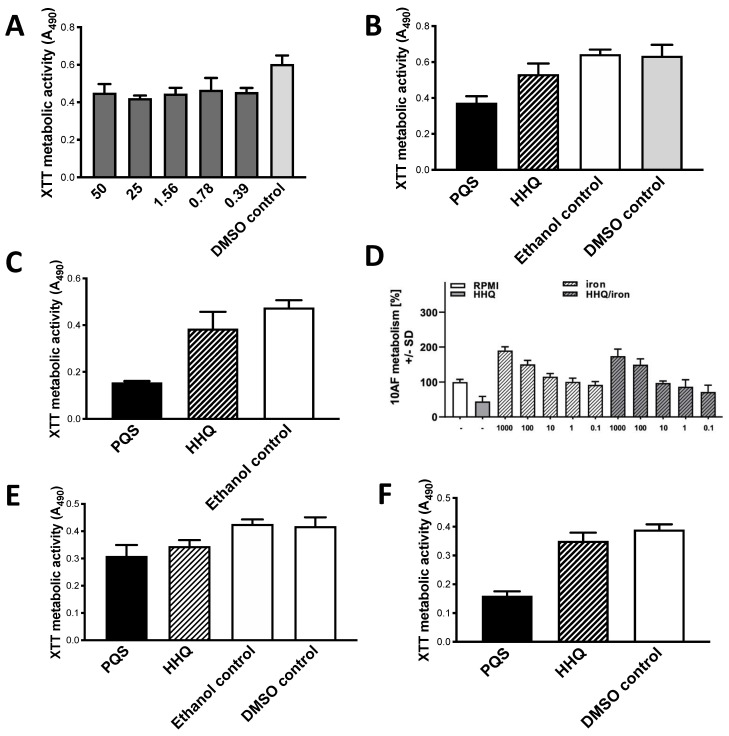
HHQ inhibits Af preformed biofilm; comparison to PQS. (**A**) Dose-response study with preformed 10AF biofilm. Flat dose-response shown. “DMSO control” is same concentration of DMSO diluent as present in test article wells. Twofold dilutions of HHQ were made from 50 to 0.39 μg/mL. Bars showing results with 3.12–12.5 μg/mL were not different from the 1.56 or 25 μg/mL bars, so are not shown in order to simplify the figure. All concentrations shown were *p* < 0.001 compared to control except 0.78 μg/mL, which was *p* < 0.01. (**B**) through (**F**): molecules tested at 1.56 μg/mL. DMSO control results same as ethanol control or RPMI alone control, in every panel. (**B**,**C**), comparison with PQS. (**B**) HHQ and PQS inhibit strain 10AF compared to respective controls, *p* < 0.01 and *p* < 0.001, respectively. PQS more potent than HHQ, *p* < 0.001. (**C**) HHQ inhibits strain Af293 by 15%, but not significantly. PQS different from HHQ and control, *p* < 0.001 both comparisons. (**D**) Reversal of inhibition by Fe, no boosting above Fe alone at high Fe concentrations, Af strain 10AF. Fe was diluted twofold from 1000 to 0.1 μM final concentrations (these are the numbers on the x axis), in presence or absence of HHQ. Data presented as % of controls. HHQ alone was inhibitory compared to control, *p* < 0.001 (left two bars). At Fe 0.1 μM + HHQ (right bar), inhibition by HHQ is partially reversed; the combination still shows inhibition compared to control (*p* < 0.05), but less inhibition (*p* < 0.05) compared to HHQ alone. Fe 1–10 μM completely reverses HHQ inhibition in combination (2nd and 3rd bars from right), these are not significantly different from control, and Fe 10 μM + HHQ is *p* < 0.05 compared to HHQ alone. Fe alone at 10 μM (5th bar from left) begins to significantly boost Af metabolism (*p* < 0.05 compared to control) and higher concentrations are *p* < 0.001 compared to control. HHQ inhibition in combinations blunts the Fe boosting about 10-fold, since it is not until the combination of HHQ with Fe 100 μM (4th bar from the right) that there is significant boosting above control (*p* < 0.01; Fe 1000 μM is *p* < 0.001 compared to control). No combination of HHQ with high concentrations of Fe is boosted above the same concentration of Fe alone. Studies in hypoxic conditions, Figure 8**E**,**F**. In 8**E**, strain 10AF, HHQ and PQS inhibit to 82% and 73% of respective controls, both *p* < 0.001; not significantly different. In 8**F**, strain Af293, HHQ and PQS inhibit to 87% and 40% of respective controls, *p* < 0.05 and *p* < 0.001, respectively. HHQ and PQS effect differ by *p* < 0.001.

Inhibition was noted at ≥0.39 μg/mL, but the dose-response curve was completely flat (Figure 8A). HHQ is a less potent inhibitor than PQS in this study (Figure 8B,C).

Fe 0.1 μM partially reverses HHQ inhibition, and Fe concentrations ≥1 μM reverse the HHQ effect (Figure 8D). At high Fe concentrations, where Af metabolism is boosted, the combination of HHQ with Fe does not boost Af metabolism above that of Fe alone (Figure 8D). PQS is a greater inhibitor than HHQ under hypoxic conditions as was the case with the other congeners (Figure 8E,F).

### 3.5. Study of Congeners in Growth Inhibition

**Figure 9:**
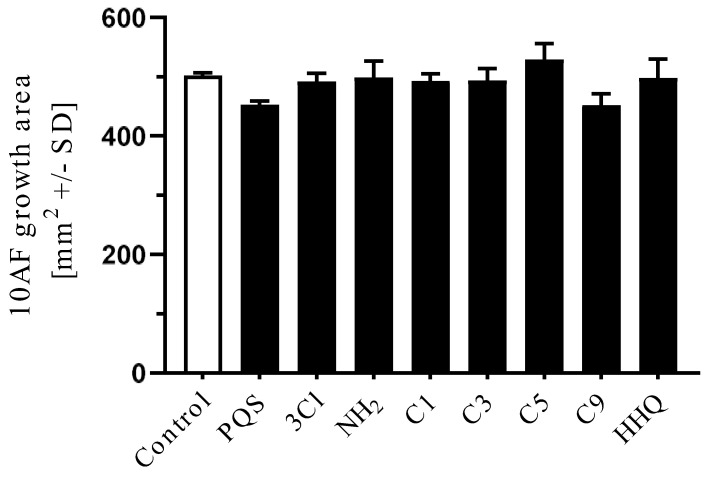
PQS and C9-PQS inhibit 10AF growth on agar. Effects of PQS, 3Cl-PQS (3Cl), NH_2_-PQS (NH_2_), HHQ, and C1-, C3-, C5-, C9-PQS were tested against 10AF growth on RPMI agar. All molecules were tested at 12.5 μg/mL. Only C9-PQS and PQS inhibited significantly (both *p* < 0.001), compared to control. PQS was more inhibitory compared to C1-PQS, C3-PQS, C5-PQS (all *p* < 0.001), and to NH_2_-PQS, 3Cl-PQS, HHQ (all *p* < 0.05). PQS and C9-PQS inhibited growth equally.

It was of interest to compare the congeners with respect to a likely more demanding consequence in intermicrobial aggression, inhibition of growth (in our studies, on agar). For this, data was already available on a PQS concentration with a marked effect [18], and at this concentration (12.5 μg/mL) all the studied molecules were compared (Figure 9).

Similar to results obtained when investigating the effects of PQS and PQS congeners on 10AF biofilms, PQS and C9-PQS showed strong effects on 10AF growth on agar, but less dramatic than those seen in biofilm studies. Antifungal effects of PQS and C9-PQS were similar. The other molecules studied were ineffective (Figure 9).

We attempted to repeat this study with all reagents studied at 100 μg/mL. We now noted PQS and C9-PQS again inhibitory (both *p* < 0.001) compared to controls, but also now 3NH_2_-PQS (*p* < 0.001) and C3-PQS (*p* < 0.01) were inhibitory, and C1-PQS, C5-PQS, and 3Cl-PQS were modestly inhibitory (*p* < 0.05). The improvement of PQS and C9-PQS, compared to that of 12.5 μg/mL was, however, small. Although we can conclude there was inhibition of Af growth, interpretation of that dose-response, as well as the possibility of a larger inhibitory amount of all the molecules tested at 100 μg/mL, cannot be excluded, because it was evident there were some solubility problems with all the test molecules at this concentration.

## 4. Discussion

In an overview of the present studies, every congener studied was similarly inhibitory to Af biofilm, with exception of C1-PQS. All the inhibitory congeners were similar in activity to each other, with a significant effect showing at approximately the 1 μg/mL range for all. This is similar to the minimum inhibitory concentration required for PQS [18], where (Figure S1) 3 μM was that concentration for preformed biofilm (Figure S1, part B) (3.85 μM PQS = 1 μg/mL). However, in every direct comparison of congeners with PQS on Af biofilm on a μg/mL basis, PQS was always the more potent inhibitor of biofilm. This suggests that the critical Af-inhibiting moeities of the PQS molecule were not completely interfered with by the molecular modifications, with the exception of an alkyl chain length <3 carbons, where the activity fell many-fold. It is of note that Pa appears to naturally produce PQS analogs with alkyl side chain lengths of 5 and 9 molecules long, in addition to PQS (7 carbon side chain) [29]. In summary, if any of the congeners studied were proven useful therapeutically, in altering Pa pathogenicity, it might be expected their effects on intermicrobial competition with Af would be similar to that of the parent PQS (but variably so, dependent on congener).

Similarly, the dose-response curves for the inhibitory congeners were all similarly flat, with concentrations many-fold higher for each adding little to the inhibitory activity. This could indicate the initial inhibition was maximal, or possibly an irreversible inactivation of the target. The dose-response curve for PQS with preformed Af biofilm was only marginally steeper [18] (Figure S1, part B). We note that if all comparisons in the present study had been made and expressed on the basis of molarity rather than μg/mL, the conclusions would be unchanged. For example, the masses (Table 1) for C1-PQS and C9-PQS, the largest mass differences, where there is less than a two-fold difference. C1-PQS was virtually noninhibitory, whereas C9-PQS was almost as potent as PQS. This example, and the flat dose response-curves for every congener, emphasizes that if comparisons had been adjusted for molarity rather than μg/mL, the conclusions about activity would be unchanged. The molarity differences for the other congeners, would be even smaller than the two examples just cited.

In yet another similarity, and recalling that RPMI-1640 medium has minimal Fe content (Methods), added Fe reversed the inhibition with every congener (as well as PQS [18]). This was noted at the low end of the added Fe concentrations studied, 0.01–1 μM Fe. In PQS studies, this reversal has been interpreted as possibly related to the known Fe chelation properties of PQS [10,11,12,13], denying Fe to Af in the milieu, but other explanations are possible. For example, the congeners may act on the Af, and interfere with some Af Fe-dependent metabolic activity directly, either related to Af Fe uptake, at the cell wall or membrane, or internal metabolism in the Af cell. In this regard, we found that one effect of PQS on Af involved Af siderophores [18], so a direct effect of PQS and related molecules on Af processes externally or internally is a distinct possibility. Another possibility is that the congeners themselves are not the actors, but rather are molecules that are processed by Af into molecules biologically active on Af; this capability has been elegantly detailed with other Pa molecules [30], and would be consistent with the observation that other micro-organisms can inactivate PQS [16]. This discussion is particularly relevant, in that some of the congeners are believed to be minimal Fe chelators on their own [10,20,31].

Another similarity among the congeners, and with PQS, is that the activities were similar in hypoxic and normoxic conditions. Action under hypoxic conditions was of interest because: (a) a hypoxic environment is relevant to areas of the lung in CF [32,33,34,35,36]; (b) Pa-Af interactions can be quite different under reduced oxygen concentrations [28]; (c) anaerobic Pa does not make PQS, owing to the oxygen dependence of the PqsH enzyme that converts HHQ into PQS [8,29]; and (d) molecules closely related to PQS, such as HHQ, may be dominant at different levels of hypoxia [29]. Our findings suggest the inhibitory activities may not be critically dependent on absolute ambient oxygen concentrations.

The biggest departure that appeared in these studies, from a property described for PQS [18], is that, unlike PQS, none of the congeners, at high Fe concentrations, boosted Af metabolism above that for Fe alone. This suggests that some property of the intact PQS molecule is required for this biologically unique microbial interaction, which was shown to require Af siderophores for maximal effect [18]. It is relevant that the structural specificity of the PQS molecule has been noted for several described PQS functions [17,20,21]. One research team noted “almost every part of the PQS molecule appears to contribute towards its function as a signal” [21]. We should note here that although there is published research on the effects, on Pa, of the molecules we studied [17,20,21], we have studied the direct effects of the molecules on Af. We have not examined the effects of the molecules on Pa, such as the nature of Pa supernatants, after exposure to these molecules, on Af. Such research is a useful future pathway.

In the present studies we did not investigate the inhibition of Af biofilm formation, except with 3NH_2_-PQS. In that study, the inhibition of Af biofilm formation was also seen, but a higher concentration of congener was required than that needed to initiate inhibition of preformed Af biofilm. This was also true with PQS [18], (Figure S1), which is noteworthy, in that in the majority of our studies of biofilms, preformed biofilms have been more resistant to various agents than forming biofilms (the latter initiated by Af conidia, whereas preformed biofilms are comprised of interlocking hyphae, with extracellular material attached [37]).

A previous study [17] examined the effect of a variety of congeners of PQS on Af, but only at a single concentration of 100 μM each. These investigators found the active molecules prevented the Af switch from conidia to hyphae, which would make forming biofilm more susceptible to congener action than preformed biofilm. A second divergence from our observations is that examination of that article’s Supplementary Materials apparently reveals that 3Cl-PQS was inactive in their studies [17]. A possible explanation of these two divergences relates to our studying inhibition by the XTT assay, which assesses the effects of Af metabolism, whereas their study assessed biofilms by the crystal violet assay, which assesses biofilm mass, and indirectly, biofilm growth. The differences between the two studies could be expected, given that effects on metabolism would be earlier and more sensitive markers of effect, before differences in growth would be significant. However, in other parts of their studies [17] these investigators concluded that fungal growth was unaffected by the molecules, although biofilm growth was affected, the mechanism of these different conclusions being unexplained.

In our growth studies, PQS and PQS congener effects against 10AF growth on agar were consistent with effects observed for 10AF biofilms, i.e., PQS and C9-PQS showed the strongest effects. There are two explanations for the quantitative differences noted in our two types of assays. One, consistent with the concept that effects on fungal metabolism would be detectable before effects on growth inhibition would be patent, effects against growth on agar were weaker than effects against fungal biofilms (compare Figure 9 with the previous figures), manifest by the inactivity of most congeners on growth even at the same biofilm study concentrations, and also by comparing PQS effects against these two types of fungal targets at the disparate PQS concentrations studied. Second, RPMI agar, which is comprised of iron-limited RPMI medium in combination with iron-rich Bacto agar, provides more iron than RPMI liquid medium alone (as used in our biofilm studies), thus the agar would reduce all iron-denying effects. Finally, we believe life in a biofilm to be more relevant to growth of Af in lungs than colony growth on agar [37].

Other researchers have reported HHQ inhibits *Candida albicans* biofilm [14]. HHQ has also been reported to have antimicrobial activity against *Staphylococccus aureus*, affecting staphylococcal fermentation pathways, and that both HHQ and 3NH_2_-PQS suppress *S. aureus* in a restricted Fe environment, analogous to the Fe conditions in the RPMI-1640 media we utilized [31]. It would be of interest to assess the performance of all of the molecules studied in an environment enriched for Fe, as other Pa molecules appear to come into play in high, compared to low, Fe milieu [38].

## Figures and Tables

**Table 1:** Alkyl quinolones.

Molecule	Molecular Weight	Molarity of 1 μg/mL Solution	Structure
C1 PQS	175.2	5.71 μM	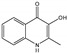
C3 PQS	203.2	4.92 μM	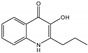
C5 PQS	231.2	4.32 μM	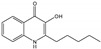
PQS (C7)	259.2	3.85 μM	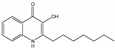
C9 PQS	287	3.48 μM	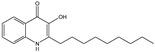
HHQ	243	4.12 μM	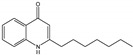
3-NH_2_-PQS	258	3.88 μM	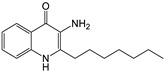
3Cl-PQS	277.8	3.6 μM	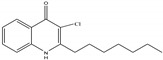

## Data Availability

Original data is available at www.cimr.org.

## Supplementary Materials

The following are available online at https://www.mdpi.com/article/10.3390/jof7050343/s1, Figure S1. PQS inhibits 10AF biofilm formation.
