# Supplementary material for: Molecular Modifications of the Pseudomonas Quinolone Signal in the Intermicrobial Competition with Aspergillus
Source: J Fungi (Basel). 2021 Apr 28;7(5):343. doi: 10.3390/jof7050343 (PMC8146305; doi:10.3390/jof7050343)
Supplement: Supplementary file 1 [file jof-07-00343-s001.zip › jof-1165460-supplementary.pdf]

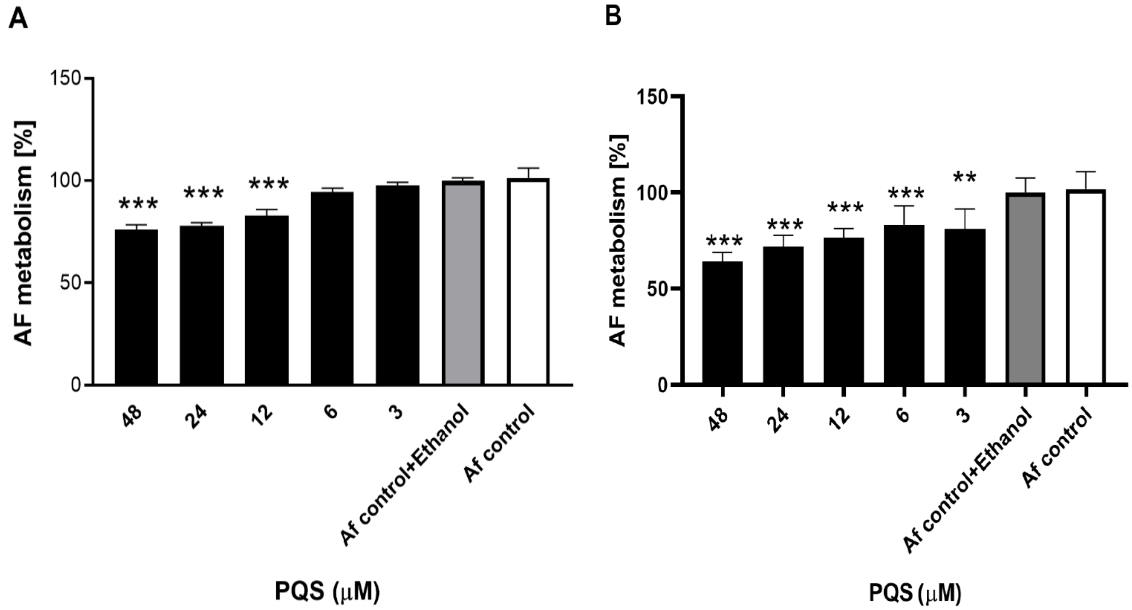

Figure S1. PQS inhibits 10AF biofilm formation. (**A**). PQS inhibits 10AF biofilm formation. 10AF forming biofilm on plastic was incubated with two-fold dilutions of PQS or a solvent control. XTT assay, eight replicates/group were studied. PQS ≥ 12 μM significantly inhibits. Three asterisks = $p < 0.001$, compared to controls. Concentrations 96–386 μM also were significant at this level (not shown), with small non-significant increases in inhibition compared to PQS 12 μM. (**B**). PQS inhibits preformed 10AF biofilm. 10AF preformed biofilm was incubated with two-fold dilutions of PQS or a solvent (ethanol) control. The x axis numbers are μM PQS, XTT assay, eight replicates. PQS 3 μM significantly inhibits, lower concentrations studied did not. Three and two asterisks = $p < 0.001$, $p < 0.01$, respectively, compared to controls. Concentrations 96–386 μM also were significant at $p < 0.001$.

With accordance of the publisher, this figure republished from Nazik, H., Sass, G., Ansari, S.R., Ertekin, R., Haas, H., Deziel, E., Stevens, D.A. Novel intermicrobial molecular interaction: Pseudomonas aeruginosa Quinolone Signal (PQS) modulates Aspergillus fumigatus response to iron. *Microbiology*. 2020;166:44–55.
